# Functional Outcome of All-Soft-Tissue Quadriceps Tendon Autograft in ACL Reconstruction in Young and Athletic Patients at a Minimum Follow-Up of 1 Year

**DOI:** 10.3390/jcm11226706

**Published:** 2022-11-12

**Authors:** Lorenz Pichler, Ludwig Pichler, Markus Liu, Stephan Payr, Harald Binder, Georg Kaiser, Marcus Hofbauer, Thomas Tiefenboeck

**Affiliations:** 1 Department of Orthopedics and Trauma-Surgery, Medical University of Vienna, 1090 Vienna, Austria; 2 Klinik Ottakring, 1160 Vienna, Austria

**Keywords:** ACL, quadriceps tendon, all soft tissue, arthroscopy, sports medicine

## Abstract

To investigate the functional outcome in young and athletic patients with ACL injuries, treated with an all-soft-tissue quadriceps tendon autograft at a minimum follow-up of 12 months. **Methods:** Patients who received a QT autograft ACL reconstruction between August 2018 and December 2020 were included in this study. Range of motion in the operated knee was described at 6 and 18 weeks after surgery and the functional outcome parameters (Lysholm score, IKDC score and Tegner activity scale) were calculated at 6 and ≥ 12 months after surgery. **Results:** Forty patients were included in this study, of which 29 identified as male and 11 as female. The average age was 31.3 years (range 16 to 57 years) and the mean follow-up time was 16.8 months (range 12 to 30 months). All functional outcome scores showed improvement over the course of the follow-up: Lysholm score 94.2 to 95.5 (n.s.), IKDC score 90.1 to 93.9 (n.s.), Tegner activity scale 3.7 to 5.0 (*p* > 0.001), all at six months and ≥12 months. No reruptures happened during the time of the follow-up. **Conclusions:** This study shows that the all-soft-tissue quadriceps tendon autograft technique can improve functional outcome in young and athletic patients with an ACL injury at short to intermediate follow-up.

## 1. Introduction

Every year, around two million anterior cruciate ligament (ACL) injuries happen worldwide [1]. The majority of patients injured are young and athletic with over 50% of cases between 15 and 25 years of age. [2]. Based on the idea to create an international consensus regarding the best available evidence on operative versus non-operative treatment of ACL injuries the Panther Symposium ACL Treatment Consensus Group was formed in 2019. Made up of 66 international experts on the management of ACL injuries the symposium voted on and subsequently published 10 statements regarding the decision for operative versus non-operative care. One of these statements recommends that in highly active patients, operative treatment should be sought, given their high risk of secondary meniscal and cartilage injuries [3].

Operating techniques and choices of graft for ACL reconstruction vary widely with the hamstring autograft (HT) being the most common, followed by bone-patellar-tendon-bone (BPTB) [4,5,6]. Both techniques however carry a certain risk for donor site morbidities, such as patella fractures in BPTB or anterior knee pain in hamstring tendon autografting [7].

A technique that has been getting more attention recently is quadriceps tendon autografting (QT). Cavaignac et al. [8] compared QT to HT and found that functional outcome (residual laxity and patient reported outcome) after 3.6 years of follow-up were equal or even better in QT-patients. Furthermore QT showed less donor site symptoms than HT and no difference in knee stability or the rate of reoperations [9]. Lower rates of donor site symptoms and equal graft failure rates, as well as stability can also be found when comparing QT to BPTB, which has been shown in a large meta-analysis [10].

Considering these findings, quadriceps tendon autografting recently became subject to more thorough scientific investigation. Krebs et al. [11] proved in a study on cadaveric specimens, that the quadriceps tendon is suitable to produce ‘a robust autograft for ACL reconstruction’. This statement was affirmed by biomechanical testing on cadaveric QT autografts which showed similar levels of functionality under loads simulating clinical examinations, compared to HT [12]. If QT reconstruction is considered by the surgeon as technique of choice, MRI can be used to assess the individual suitability of patients for the QT-technique and predict autograft size [13]. However, even if in patients where a full thickness autograft is not possible, QT remains a viable option as no difference in outcomes or complications have been reported between full thickness and partial thickness grafts [14].

While most studies on QT repair recently published, describe a technique where the tendon is harvested with a patellar bone block [15] the results presented are based on an all-soft-tissue technique. As stated earlier, the group most at risk for ACL injuries and who have the highest benefit from surgery, are the young and athletic [1,3]. This study reports functional outcome and re-rupture rates in patients after a QT ACL repair with a mean age of 30 years and a primary injury sustained during sports.

The aim of the study is to carry out a retrospective analysis of the progression of well-established functional outcome scores over the course of 12 months after ACL reconstruction with a QT autograft. The hypothesis put to test is that patients treated with an all-soft-tissue quadriceps tendon autograft ACL reconstruction, showed improvement in the outcome scores under investigation during a 6 and ≥12 month post-operative follow-up.

## 2. Material & Methods

### 2.1. Patients

An ethics approval by the local ethics committee was retrieved and a retrospective analysis of patient cases with anterior cruciate ligament reconstruction via all-soft-tissue quadriceps tendon autograft was carried out. Initial selection criteria for the reconstruction via a QT-autograft were defined as follows: primary reconstruction, high functional demand, no preexisting injuries to the quadriceps tendon or the patella and a minimum follow-up of 12 months. 52 patients who underwent reconstruction via a QT-autograft during the investigated period were found. 12 cases presented as re-ruptures of already reconstructed ACLs and were therefore excluded. 40 Patients (29 male, 11 female) between 16 and 57 years of age, with a mean age of 31.3 years, matched all the inclusion criteria, including the minimum follow-up and were therefore included. Included patients had a clinically and MRI confirmed complete rupture of the ACL. Exclusion criteria were defined as follows: additional collateral ligament injuries requiring surgical intervention or chondral injuries classified as >2 using the Outerbridge classification.

All injuries happened during sports.

### 2.2. Surgical Technique

Reconstructions were carried out between 2018 and 2020, at a university hospital by three surgeons highly trained on QT ACL repairs or under their supervision.

All surgeries took place under general anesthesia and with the use of a tourniquet. At first, a diagnostic arthroscopy was carried out during which the following structures were examined for injury: suprapatellar recess, retropatellar cartilage, medial and lateral joint compartment, including both menisci, femoral and tibial cartilage. In cases where a meniscal tear was found it was addressed either through a partial meniscectomy or suture, depending on its location. No further stabilization via lateral extraarticular tenodesis was performed.

**Figure 1:**
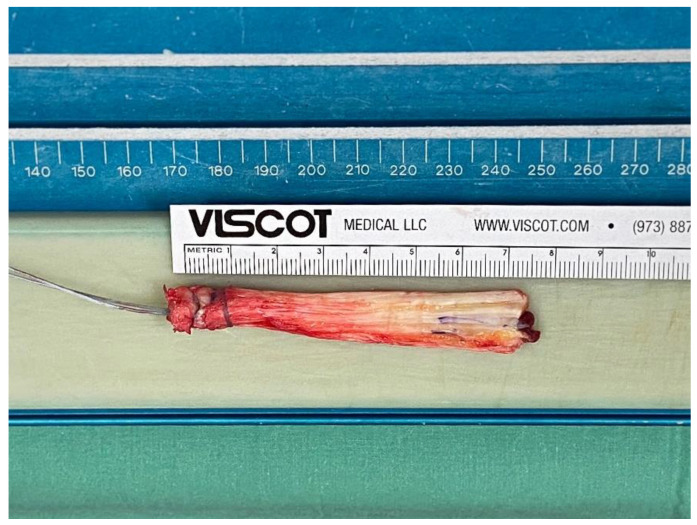
All soft tissue quadriceps tendon autograft immediately after harvesting.

Following arthroscopy, the all-soft-tissue quadriceps tendon autograft (Figure 1) was retrieved through a longitudinal incision, 1 cm proximal to the cranial patella. The autograft was harvested from the medial aspect of the tendon, utilizing a vertical parallel cutting tendon knife. It was then installed in the injured knee using an anatomic ACL reconstruction technique utilizing a TightRope RT system (Arthrex, Naples) with a button on the femoral side and a biocomposite interference screw on the tibial side [16]. Both sockets, the femoral and tibial, were drilled through the corresponding footprint. Anatomical reconstruction was confirmed via intraoperative X-ray, as well as arthroscopically and an intraarticular drainage was put in place. The application of an elastic bandage and a rigid knee brace ended the operation.

### 2.3. Post-Operative Procedure & Assessment

Postoperative care and remobilization differed between patients according to whether an accompanying meniscal injury was treated with refixation and if so, whether it was a medial or lateral meniscal injury (Table 1).

Patients underwent standard post-operative clinical knee assessment, including Lachman and anterior drawer test and documentation of range of motion (ROM) at 6 and 18 weeks after surgery. Additionally the following well stablished functional outcome scores have been used to assess patients at 6 and ≥12 months follow-up: Lysholm knee scoring scale (LKSS), Tegner activity scale (TAS), International Knee Documentation Committee Subjective Knee evaluation form (IKDC). The pain level was assessed using the Visual Analoge Scale (VAS), utilizing a numerical rating scale from 1 to 10 on a conventional VAS ruler at 12 month follow-up.

### 2.4. Statistics

Based upon demographic variables (sex, age and follow-up), a statistical analysis of functional outcome over time was completed. Descriptive values for all patients (median, mean, range and proportions) were analyzed and tested for the differences using unpaired students t-tests with a *p*-value of 0.05, set as statistically significant. Calculations were carried out using Microsoft Excel Version 16 and SPSS Version 26.

## 3. Results

With 40 patients undergoing quadriceps tendon autograft ACL repair, a follow-up of 12 months or more was achieved, the mean follow-up being 16.8 months (range 12 to 30 months).

Mean patient age was 31.3 years (range 16 to 57 years), with 29 (73%) patients identifying as male and 11 (27%) as female. Almost all injuries occurred during sports with football being the most common cause (13 cases, 33%). 12 cases of reruptures of previously treated ACL injuries found during the research for this study were not included (Table 2).

Surgery was performed at 2 weeks after trauma, in cases with accompanying knee injury, such as a meniscal tear applicable for suture and post-trauma range of motion of at least S 0-0-90°. All other cases were treated after the initial inflammation phase and preoperative physiotherapy at 6 weeks post trauma. Applying this scheme, the average time to surgery was 2.5 months after injury (range 0.1 to 10.4 months).

In 19 (48%) cases, additional meniscal tears were found of which, 8 (42%) were addressed with refixation through sutures and 11 (58%) with partial meniscectomy. Medial collateral ligament lesions were diagnosed in 2 (5%) patients, all of whom were treated conservatively before ACL repair. Intraoperative MCL stability testing showed no remaining signs of instability in these patients.

There were no cases of reruptures in patients included in this study during the time of the follow-up. Pain and hypaesthesia around the graft harvesting area at ≥12 month follow-up was reported by 2 (5%) of patients. Topical vancomycin was applied to all autografts during surgery and there were no surgical infections whatsoever.

Comparison of the functional outcome scores showed that all scores improved over the course of the follow-up with IKDC score, TAS and range of motion progress, reaching statistical significance (Table 3). Range of motion enhanced from an average of 108° flexion (range 40°–150°) at 6 weeks to 131° flexion (range 90°–150°) at 18 weeks after surgery, reaching statistical significance as well. All patients showed full extension at 6 and 18 weeks of follow-up and improvements in flexion or maintenance of their already full ROM from six to 18 weeks. Clinical assessment, including Lachman and anterior drawer test showed sufficient knee stability in all patients at 6 and 18 weeks.

## 4. Discussion

This study showed that the all-soft-tissue quadriceps tendon autograft technique can provide improved functional outcome in patients with ACL injuries at early to intermediate follow-up. Mouarbes et al. [17], in their large scale systematic review, attested the QT-technique a functional outcome that is on par with BTPB and slightly superior to HT. Their reported Lysholm score of 90.7 (range 90.6–90.99) and IKDC score of 83.1 (range 82.6–83.7) were exceeded by those of the present study with Lysholm scores of 94.2 to 95.5 and IKDC scores of 90.1 to 93.9, both at 6 to ≥12 months follow-up. A reason for this could be found in the young and very sportive nature of the patient collective, upon which this work is based. The significant improvement of the Tegner activity scale (3.7 to 5.0, 6 to ≥12 months) underlines this and shows that the all-soft-tissue QT autograft is suitable to bring young athletic patients back to sports within a short time frame—a conclusion that is in line with other studies on the QT autograft technique in athletes [18] but with limitations to it, as patients only received full clearance for high level activities at 9 months post-operatively.

Concerning graft failures, the QT-technique was attested a rerupture rate of 4.7% by Lind et al. [19]—a finding which could not be confirmed by our results (0 reruptures). An explanation for this discrepancy might have been given in another publication by Lind et al. [20], where the authors state that the failure rate of QT autografts for ACL repair differs significantly between clinics with a high volume of procedures taken out (2.9% revisions) and those with lower operating numbers (6.4% revisions). In the case of the present study, all patients have been treated by three surgeons highly trained in the QT-technique or under their direct supervision.

With an average follow-up of 16.8 months and 0 reruptures, the present study also stands in contrast to reruptures rate of up to 10.7% at long-term follow up of 5 years [21]. A hypothesis for this difference in low to mid-term failure rates, as found by us, to higher long-term failure rates, as quoted in literature, was put up by Haybäck et al. [22]. Haybäck suspected the incidence of QT reruptures increased disproportionally over time. Light should be shed on this suspicion over the next couple of years as the QT autograft becomes more popular, not only in revision surgery, and more long-term follow up data should become available.

The suitability of the QT autograft as the technique of choice in revision surgery has been investigated by Hunnicutt et al. [23]. The authors reported a graft failure rate of 13.8%, among all cases with an average time to failure of 26.5 months. Revision cases were not included in this study and only 12 cases were found during the research. This only allows for a very limited conclusion on this subject. The only incidence of a rerupture among cases reviewed for inclusion happened in a revision case at 6.1 months after surgery (8.3% graft failure rate among revision cases found). The average follow up on all revision cases was 17 months. While the usage of a QT autograft as technique of choice is already quite common in revision cases [24], its role as a primary graft has been described less—a novelty of this study.

Lower rates of donor site pain, compared to other techniques is another advantage of the QT autograft that has been described before [8] and which could be underlined with the data presented (2 patients (5%) with donor site pain at ≥12 months).

As a synopsis of all these findings described, the QT autograft presents as a viable option in ACL repairs, regardless of patients’ physical constitution [25]. Its favorable outcome however might also depend on the skill level of the surgeon applying it [20]. With first studies on the further development of this technique towards an even less invasive procedure [26], future research should focus on the technical evolution of this promising procedure.

### Limitations

As a retrospective analysis without a control group this study carries the limitations of any study with said study design. There is a certain risk for bias when including cases with multiple knee pathologies, however cases of isolated ACL injury are very rare and those with multiple pathologies can be considered the norm. There was no data on preoperative functional knee scores in patients who underwent surgery, so no conclusion on improvement from individual baseline can be made.

## 5. Conclusions

This study shows that the quadriceps tendon autograft technique for ACL repair can improve functional outcome in young and athletic patients with ACL rupture at short to intermediate follow-up. It was proven that with this technique, a low to non-rate of reruptures can be achieved. The results on donor site pain presented further emphasis the benefit of patient comfort of the QT autograft. Future research should focus on the evolution of this promising technique.

## Figures and Tables

**Table 1:** Postoperative care and remobilization.

Operative Procedure	Week 1–2	Week 2–4	Week 4–6	3 Months	9 Months
ACL reconstruction + partial meniscectomy or no meniscal procedure	Rigid knee brace	Rigid knee brace	Rigid knee brace	Return to running and biking	Return to pivoting sports in regard to physio-therapeutic testing
	ROM 0-0-60°	ROM 0-0-90°	ROM 0-0-90°		
	partial weight bearing (20 kg)	full weight bearing	full weight bearing		
	CPM & weekly physiotherapy				
ACL reconstruction + lateral refixation	Rigid knee brace	Rigid knee brace	Rigid knee brace	Return to running and biking	Return to pivoting sports in regard to physio-therapeutic testing
	ROM 0-0-40°	ROM 0-0-60°	ROM 0-0-60°		
	no weight bearing	no weight bearing	no weight bearing		
		CPM & weekly physiotherapy	CPM & weekly physiotherapy		
ACL reconstruction + medial refixation	Rigid knee brace	Rigid knee brace	Rigid knee brace	Return to running and biking	Return to pivoting sports in regard to physio-therapeutic testing
	ROM 0-0-60°	ROM 0-0-60°	ROM 0-0-90°		
	no weight bearing	partial weight bearing (20 kg)	partial weight bearing (20 kg)		
		CPM & weekly physiotherapy	CPM & weekly physiotherapy		

**Table 2:** Patient demographics.

Variables	*n*
Number of patients	40
Follow-up, mean, months	16.8 (range 12–30)
Sex	
Male	29 (73%)
Female	11 (27%)
Age, years	31.3 (range 16–57)
BMI, kg/m^2^	24.9 (range 20.8–31.3)
Initial revisions, excluded in study	12
Meniscal tear	19 (48%)
Meniscal tear treatment	
Suture	8 (42%)
Partial meniscectomy	11 (58%)
Medial collateral ligament lesions	2 (5%)
Graft harvesting site pain at 12 months	2 (5%)

**Table 3:** Functional outcome scores.

Functional Outcome Score Mean (Median, Range)	6 Months	≥12 Months	*p*-Value
Lysholm score	94.2 (98.0, 60.0–100.0)	95.5 (100.0, 50.0–100.0)	n.s.
IKDC score	90.1 (95.0, 59.0–100.0)	93.9 (100.0, 40.0–100.0)	n.s.
Tegner activity scale	3.7 (4.0, 2.0–4.0)	5.0 (4.5, 3.0–8.0)	<0.001
VAS, mean		0.4 (0.0, 0.0–5.0)	

## Data Availability

The data presented in this study is available upon request from the corresponding author.
